# Active nitrogen fixation by *Crocosphaera* expands their niche despite the presence of ammonium – A case study

**DOI:** 10.1038/s41598-019-51378-4

**Published:** 2019-10-21

**Authors:** Keisuke Inomura, Takako Masuda, Julia M. Gauglitz

**Affiliations:** 1 School of Oceanography, University of Washington, Seattle, Washington USA; 2 Institute of Microbiology, The Czech Academy of Sciences, Opatovický mlýn, Třeboň, Czech Republic; 3 Global Change Research Institute, The Czech Academy of Sciences, Drásov, Czech Republic; 4 Collaborative Mass Spectrometry Innovation Center, Skaggs School of Pharmacy and Pharmaceutical Sciences, University of California, San Diego, USA

**Keywords:** Ecological modelling, Microbial ecology, Element cycles

## Abstract

Unicellular nitrogen fixer *Crocosphaera* contributes substantially to nitrogen fixation in oligotrophic subtropical gyres. They fix nitrogen even when significant amounts of ammonium are available. This has been puzzling since fixing nitrogen is energetically inefficient compared with using available ammonium. Here we show that by fixing nitrogen, *Crocosphaera* can increase their population and expand their niche despite the presence of ammonium. We have developed a simple but mechanistic model of *Crocosphaera* based on their growth in steady state culture. The model shows that the growth of *Crocosphaera* can become nitrogen limited despite their capability to fix nitrogen. When they fix nitrogen, the population increases by up to 78% relative to the case without nitrogen fixation. When we simulate a simple ecological situation where *Crocosphaera* exists with non-nitrogen-fixing phytoplankton, the relative abundance of *Crocosphaera* increases with nitrogen fixation, while the population of non-nitrogen-fixing phytoplankton decreases since a larger fraction of fixed nitrogen is consumed by *Crocosphaera*. Our study quantitatively supports the benefit of nitrogen fixation despite the high electron/energy costs, even when an energetically efficient alternative is available. It demonstrates a competitive aspect of *Crocosphaera*, permitting them to be regionally significant nitrogen fixers.

## Introduction

*Crocosphaera* is a major unicellular nitrogen-fixer in the ocean^1–3^ and widely used for laboratory studies^4–8^. The process of nitrogen fixation provides fixed nitrogen to themselves and the environment, supporting their growth and balancing the nitrogen budget in the ocean^9,10^. On the other hand, *Crocosphaera* can be a consumer of fixed nitrogen such as ammonium^11–14^. Culture studies have shown that *Crocosphaera* actively consumes available fixed nitrogen in batch (dynamic) cultures^11,12^ and continuous (steady-state) cultures^14^. In general, nitrogen fixers seem to prioritize using fixed nitrogen by inhibiting nitrogen fixation^15,16^. Consuming external fixed nitrogen is advantageous as it bypasses the high energy and electron utilization costs that accompany nitrogen fixation^17–19^. However, despite the availability of fixed forms of nitrogen, nitrogen fixers are observed to fix nitrogen, which would decrease their growth efficiency (here in terms of C)^18,19^. Empirical evidence shows that both nitrogen fixation as well as the utilization of organic/fixed nitrogen occur concomitantly^12,14,20^. When two competing strategies are possible, there must be implied trade-offs dictating the balance between the two. A recently developed coarse-grained model of a nitrogen fixer^19^ shows that also using ammonium will expand their niche compared to only fixing nitrogen. Conversely, the purpose of continuously fixing nitrogen under the presence of fixed nitrogen has not been elucidated. Here we focus on this other side of the question: the effect of fixing nitrogen despite the presence of ammonium.

**Figure 1:**
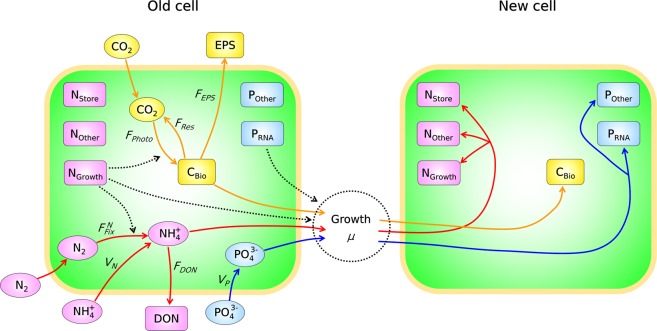
Schematics of the model. The green area represents cytoplasmic space, and the cream edge represents the cell membranes. Ovals and rectangular boxes represent inorganic and organic molecules, respectively. Different colors are applied to different elements; yellow, C; pink, N; blue, P. Solid arrows are the elemental fluxes; yellow, C; red, N; blue, P. Black dotted arrows represent positive influences. The black dotted circle represents biosynthesis. *C*_*Bio*_, cellular biomass carbon; *EPS*, extracellular polymeric substances; *P*_*RNA*_, P in RNA; *P*_*Other*_, P in other molecular pools; DON, dissolved organic nitrogen; *N*_*Store*_, N storage; *N*_*Growth*_, N in growth related molecules; *N*_*Other*_, N in other molecules. Fluxes: *μ*, growth; *F*_*Photo*_, photosynthesis; *F*_*Res*_, respiration; *F*_*EPS*_, EPS excretion; ${F}_{Fix}^{N}$, nitrogen fixation; *F*_*DON*_, DON excretion; *V*_*N*_, fixed N uptake; *V*_*P*_ fixed P uptake. The notations are same as those in Methods and Supplementary Methods.

To numerically examine this question, we have developed a quantitative model for *Crocosphaera* (Cell Flux Model of *Crocosphaera* 2: CFM-Croco2), resolving a simple set of molecular pools and minimum representation of elemental fluxes (Fig. 1) (see Methods and Supplementary Methods for details). Although we do not resolve a complex network of metabolisms as in Flux Balance Analysis^21,22^, we resolve essential metabolisms such as nitrogen fixation, nutrient uptake, respiration, photosynthesis (C fixation) and growth (Fig. 1), following previous models^19,23–25^. The strength of this minimum model is to keep the model efficient and transparent and minimize overlapping metabolic effects. Also, the development process of such minimum models often suggests missing pieces when the model does not reproduce the data. In such a case, we consider what components of metabolism would further improve the model-data fit, based on the current state of knowledge of biochemical pathways. The model results reveal that nitrogen fixation, despite the energy expenditure and allocation of resources, gives *Crocosphaera* a competitive advantage in both monoculture and in a complex community.

## Results

### Simulating steady state culture

**Figure 2:**
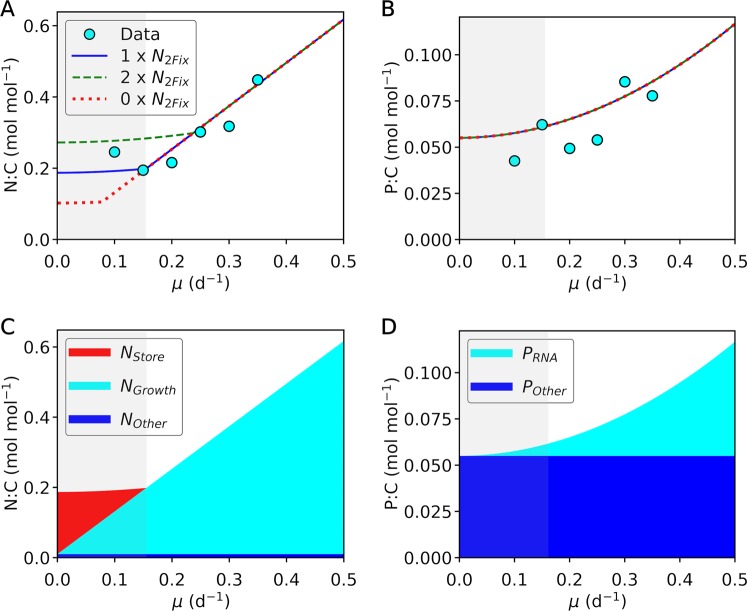
Model data comparison of N:C and P:C of *Crocosphaera* culture for multiple scenarios. (**A**) N:C. (**B**) P:C. Points are the data from the experiment^14^. The data of N:C are based on (Total N – DON – NH_4_^+^)/POC. The data of P:C are based on POP/POC. Bacterial contamination was negligible^14^. Blue curves, default run of the model manually fitted to the data. Green curves, model run with doubled nitrogen fixation. Red curves, model run with zero nitrogen fixation. (**C**,**D**) Allocation of nitrogen and phosphorus to different functionalities. Gray shadings indicate P limitation in the default nitrogen fixation; white areas are N limitation. Note: DON, dissolved organic N; POC, particulate organic C; POP, particulate organic P.

**Figure 3:**
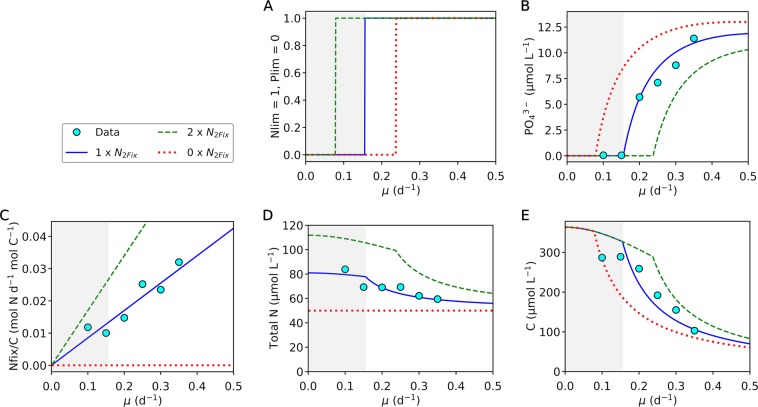
Model data comparison for different nitrogen fixing capacities. (**A**) Nutrient limitation (**B**) PO_4_^3−^ concentrations. (**C**) Nitrogen fixation rates (normalized by POC). (**D**) Total nitrogen concentration. (**E**) Biomass carbon concentration. The upper left legend applies to all the figure panels. Points represent the data from the experiment^14^. The nitrogen fixation data are based on *μ* and the concentration difference between total N and ammonium in the incoming media. Blue curves, default run of the model manually fitted to the data. Green curves, model run with doubled nitrogen fixation. Red curves, model run with zero nitrogen fixation. Gray shadings indicate P limitation with default nitrogen fixation; white areas are N limitation.

Our model reproduces laboratory data of *Crocosphaera* grown in Chemostat culture^14^. The incoming medium has 50 µM ammonium and 20 µM phosphate, and the model simulates these nutrient influxes. These macronutrient concentrations in the media are relatively high. However, the ammonium and phosphate concentrations in the culture are maintained at nanomolar-scale due to cellular uptake, when these nutrients are limiting cellular growth. The model captures the growth dependence of elemental stoichiometry of *Crocosphaera*, rate of nitrogen fixation, nutrient limitation, and standing stock of N and C (Figs 2 and 3). We further simulated scenarios with doubled nitrogen fixation and with zero nitrogen fixation. The results show increased biomass concentration with nitrogen fixation (Fig. 3E). As the growth rate *μ* increases, N:C and P:C increase due to investment for growth related molecules (*N*_*Growth*_ and *P*_*RNA*_ Fig. 2). The model captures the transition of P limitation to N limitation (Fig. 3A); in the laboratory experiment^14^ this was observed by a sudden increase in phosphate concentration when the culture became N limited from P limited (Fig. 3B). Here limitation is defined based on which nutrient controls the standing stock of biomass; i.e. if adding N increases biomass, the culture is N limited.

The model predicts that the ammonium is fully consumed, even when P is limited, due to the luxury uptake of N for N storage. This resonates with the experimental data, where the ammonium was observed at nanomolar concentrations. The model indicates that *Crocosphaera* do not accumulate quantitatively significant P in the cell based on the excess P, but they do store extra N (Fig. 2C,D), keeping the free intracellular N concentration small. The lack of P storage is inferred, as inclusion of P storage in the model was unnecessary to fit the data. In particular, the data do not show a significant change in the trend with a change from P limitation to N limitation, and *P*_*RNA*_ was sufficient to express the data. It has been shown that *Crocosphaera* produce cyanophycin^4,6^ and this result corroborates such storing capacity.

With increasing growth rate, *N*_*Growth*_ (growth related protein) increases (Fig. 2C). This leads to increases in nitrogen fixation with increased growth rate (Fig. 3C). Despite such changes, the total nitrogen in the culture decreases, since cells are flushed away at a higher rate (Fig. 3D). Total C decreases more strongly with the growth rate (Fig. 3E) due to increasing N:C of the cells.

A doubled rate of nitrogen fixation increases the nitrogen storage resulting in higher N:C under P limitation (Fig. 2A). Under N limitation, N:C is unchanged. Instead, the cellular density is increased, leading to higher total N and C (Fig. 3D,E). Also, notably, it shifts the range of limitation (Fig. 3A); e.g. N limitation is above ~ 0.15 (d^−1^) for the default run, but above ~0.23 (d^−1^) for the doubled nitrogen fixation. This indicates that the range of N limitation is narrowed down due to nitrogen fixation. Biomass C increases at growth rates where this shift occurs, but it stays unchanged where it is originally P limited. When nitrogen fixation does not occur, cells can still grow using the available ammonium. However, the stored N decreases under P limitation leading to lower N:C (Fig. 2A). Under N limitation, without nitrogen fixation, the total population decreases leading to lower biomass C in the culture (Fig. 3E).

### Simulating a simple dynamic ecological model

**Figure 4:**
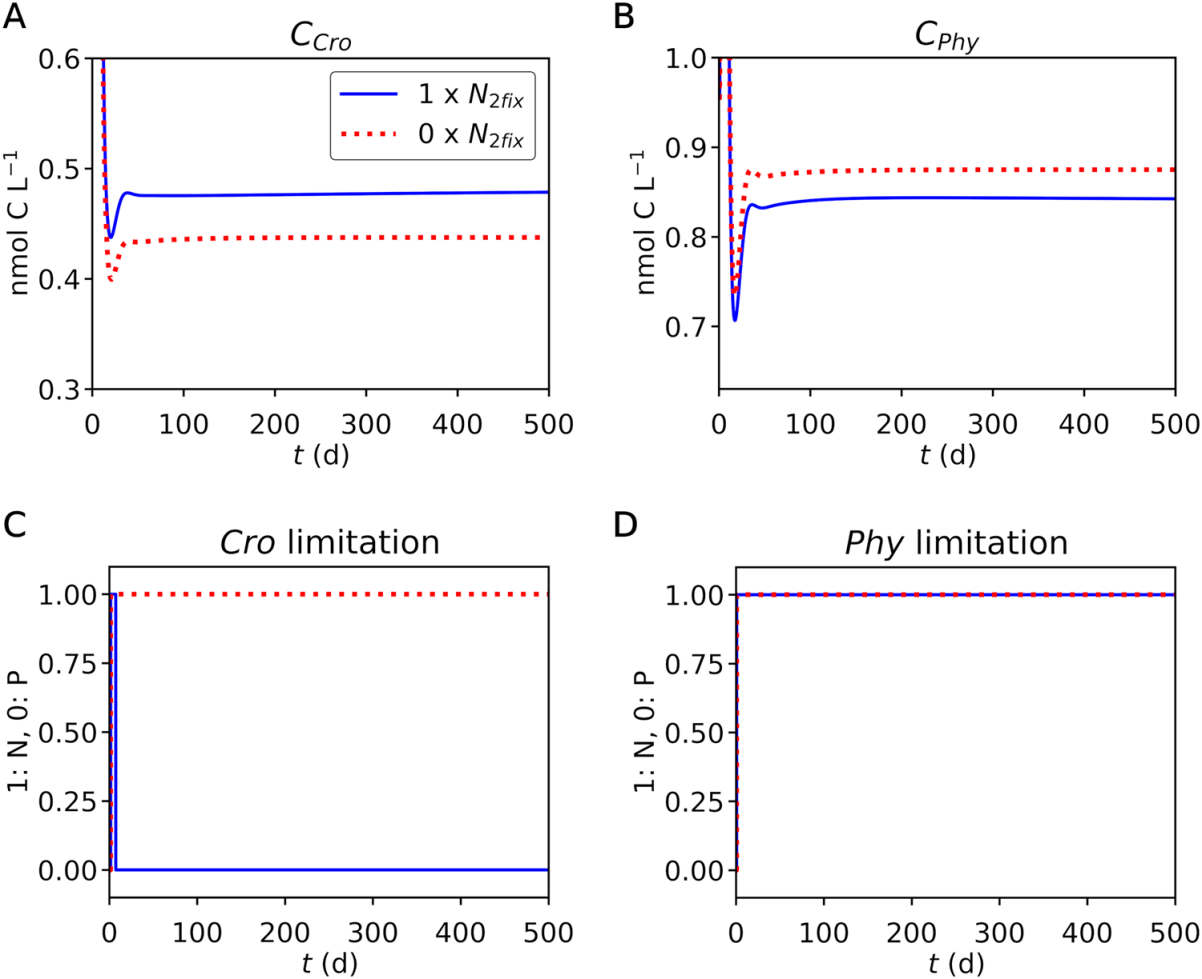
Simulated co-existence of *Crocosphaera* and non-nitrogen-fixing phytoplankton. (**A**,**B**) Concentrations of Biomass C of *Crocosphaera* and non-nitrogen-fixing phytoplankton, respectively. (**C**,**D**) Nutrient limitation of *Crocosphaera* and non-nitrogen-fixing phytoplankton, respectively. The legend in (**A**) applies to all the figure panels. Blue curves are the default run with nitrogen fixation. Red dotted curves are the run without nitrogen fixation.

**Figure 5:**
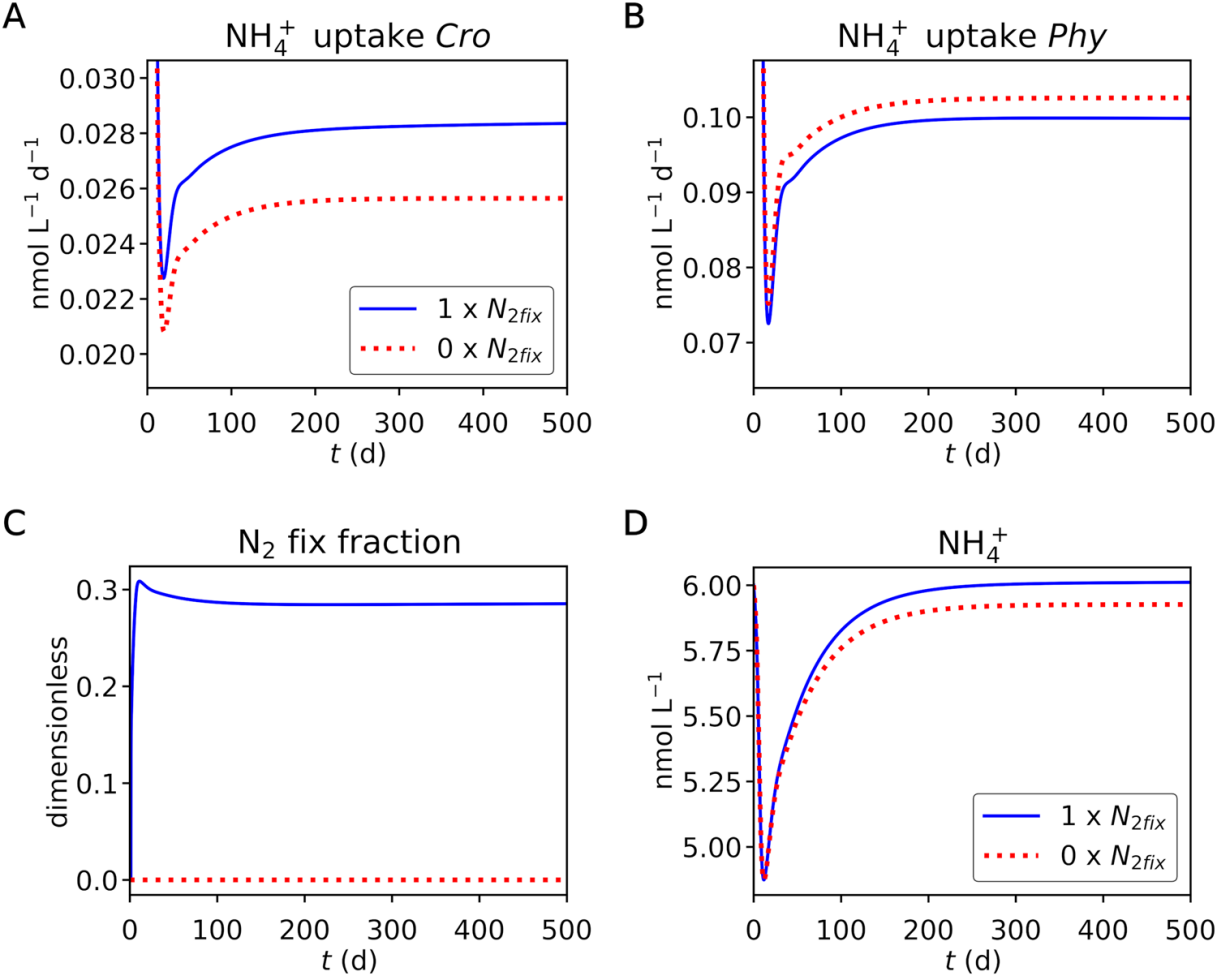
Ammonium uptake, fraction of nitrogen fixation and ammonium concentrations. (**A**,**B**) N uptake by *Crocosphaera* and non-nitrogen-fixing phytoplankton respectively. (**C**) Fraction of nitrogen fixation of all the N sources for *Crocosphaera*. (**D**) Ammonium concentration. The legend in (**A**) applies to all the figure panels. Blue curves are the default run with nitrogen fixation. Red dotted curves are the run without nitrogen fixation.

By using a set of parameters obtained from the steady state simulation, we run a simple ecosystem model (Figs 4, 5, S1 for up to day 500 and Fig. S2 for up to day 1000). Here we simulate additional non-nitrogen-fixing phytoplankton and zooplankton to represent a minimum ecosystem as used in resource competition theory^26–29^. We have prescribed higher nutrient uptake for non-nitrogen-fixing phytoplankton to give them an advantage to compensate for the lack of nitrogen fixation as conventionally modeled^27–31^. Biomass C became stable at approximately day 40 (Fig. 4A,B), where *Crocosphaera* is limited by P and non-nitrogen-fixing phytoplankton are limited by N, representing a common situation in the open ocean (Fig. 4C,D) where nitrogen fixers and non-nitrogen fixers co-exist^27–29^. The growth rate at day 500 was 0.217 and 0.382 for *Crocosphaera* and non-nitrogen-fixing phytoplankton respectively (Fig. S1), which are within the observation range^32–35^. The ratio of these growth rates is 1.76, a value similar to those previously parameterized in global ecosystem models^31,36,37^.

To test the effect of nitrogen fixation, we turned off nitrogen fixation of *Crocosphaera*. The model predicts a larger population of *Crocosphaera* when nitrogen fixation is occurring (Fig. 4A). Interestingly, the model also shows decreasing non-nitrogen-fixing phytoplankton when we allow active nitrogen fixation by *Crocosphaera*. With nitrogen fixation, we predict higher total uptake of N by *Crocosphaera* as a community (Fig. 5A), due to increased population/biomass (Fig. 4A). As a result, the N available for non-nitrogen-fixing phytoplankton decreases, lowering their population (Fig. 4B) and the amount of total N they take up (Fig. 5B).

The model predicts a relatively low fraction of nitrogen fixation (~30%) (Fig. 5C), despite low concentrations of available ammonium (Fig. 5D). These concentrations resemble those under N limitation^14^. Under laboratory conditions, *Crocosphaera* is known to grow diazotrophically when there is no added nitrogen. Thus, the result of this low fraction of nitrogen fixation is likely due to continuous addition of nitrogen to the system. Such situations may be common in the marine environment due to continuous remineralization via the microbial loop^38^.

## Discussion

### Competitive view of *Crocosphaera*

**Figure 6:**
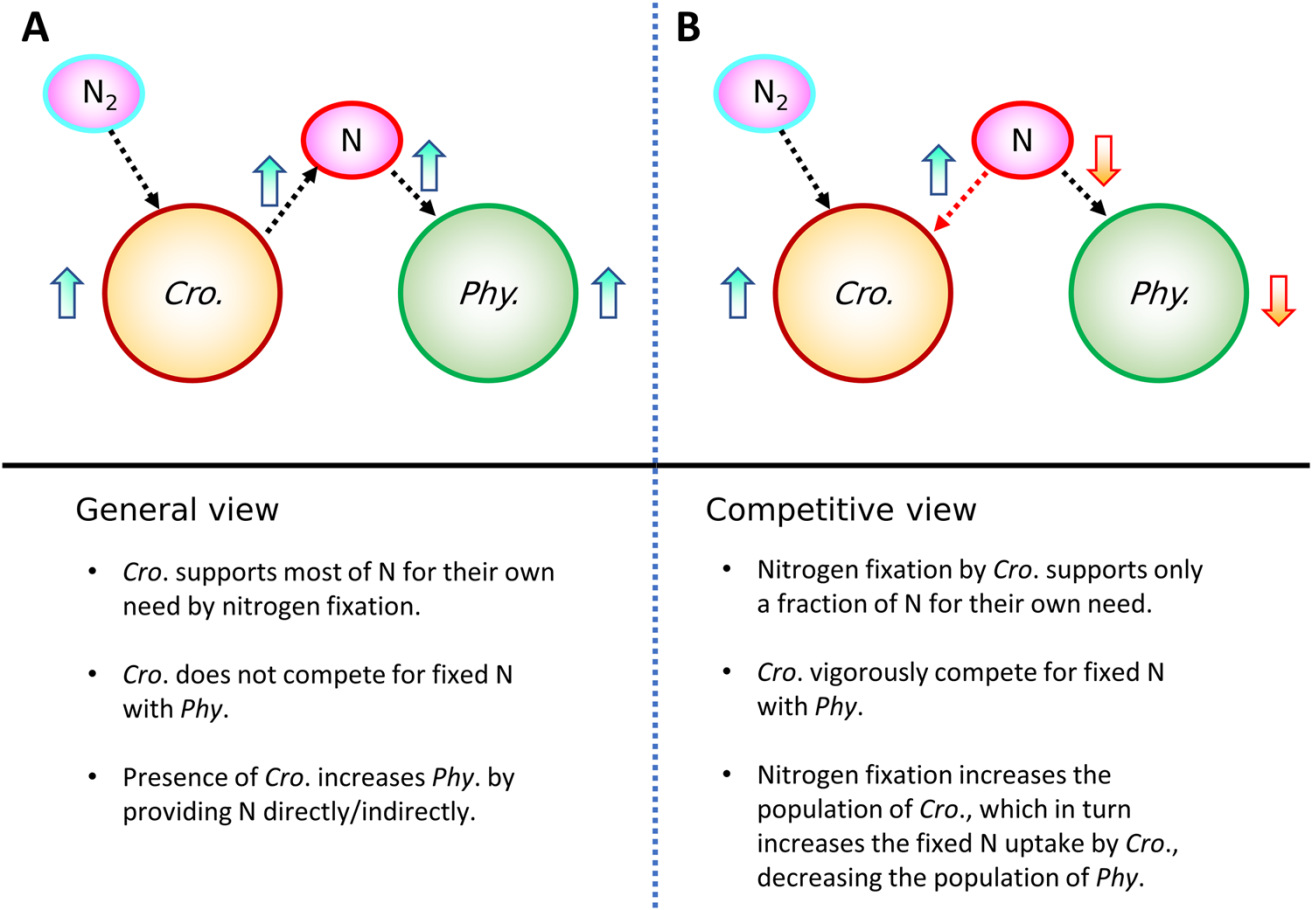
An emerged competitive view of *Crocosphaera* based on this study. (**A**) A general long-time view and (**B**) proposed short-time competitive view of how nitrogen fixation by *Crocosphaera* influences N fluxes and plankton population within a short time scale. *Cro*., *Crocosphaera*; *Phy*., non-nitrogen-fixing phytoplankton; N, fixed nitrogen; dotted arrows, fluxes; thick arrows, influence of nitrogen fixation. The differences are in red in (**B**).

Nitrogen fixers are often described as a provider for the environment since they provide fixed nitrogen to other organisms^39,40^. It is true that nitrogen fixation is essential in balancing lost fixed nitrogen^9^ and in a relatively long time scale, nitrogen fixation supports the community by providing bioavailable nitrogen^41^. Also, it is true that nitrogen fixers can grow by themselves by only using dinitrogen^6,14,42^ and excretion of N containing molecules is observed (25% ~ 50% of fixed nitrogen)^39,43^. These facts often leave an impression that nitrogen fixers actively stimulate the growth of other phytoplankton by providing fixed nitrogen (Fig. 6A). However, our study shows that nitrogen fixers can also be nitrogen limited (Fig. 3) and, within a short time scale and distance, compete with other non-nitrogen-fixing phytoplankton for fixed nitrogen (Fig. 6B). The nitrogen limitation of *Crocosphaera* is supported by nitrogen depletion in culture^14^ and our prediction of increased cellular biomass with nitrogen fixation (Fig. 3D,E).

In nitrogen limiting environments, *Crocosphaera* increases their population with nitrogen fixation, until the population becomes limited by another nutrient (here phosphorus). This increases the community uptake of nitrogen by *Crocosphaera*, limiting the nitrogen sources in the environment, and ultimately decreasing the population of other non-nitrogen-fixing phytoplankton (Fig. 6B). Since this effect might be overcome with excretion, we tested excreting 50% of fixed nitrogen to the environment. This makes the growth of *Crocosphaera* N limited and the growth rate is decreased, which was accompanied by decreased *N*_*Growth*_ and *P*_*RNA*_. However, the model shows that the population of non-nitrogen-fixing phytoplankton is still lower than in the case without nitrogen fixation. Also, we have tested a maximum uptake rate of non-nitrogen-fixing phytoplankton 10 times higher than that of *Crocosphaera*, which resulted in a growth rate for non-nitrogen-fixing phytoplankton of ~6.5 times higher than *Crocosphaera*. However, if we allow *Crocosphaera’s* nitrogen fixation, the population of non-nitrogen-fixing phytoplankton was still lower than the case with zero nitrogen fixation.

The predicted fraction of nitrogen fixation of 30% is rather low given that *Crocosphaera* can grow diazotrophically. Since this value is based on a single chemostat experiment with a fixed resource concentration of ammonium, chemostat cultures of *Crocosphaera* with various resource concentrations of fixed nitrogen might be useful to test our prediction. However, recent observations show little link between the presence of *Crocosphaera* and primary productivity^44^, supporting the presented competitive view. We note that there are multiple sources of fixed nitrogen even in the oligotrophic gyres where *Crocosphaera* is observed: atmospheric deposition^45,46^, active remineralization by members of the microbial loop^38^ and occasional upwelling^47^. Such N sources may decrease the fraction of nitrogen fixation, making *Crocosphaera* competitive. Also, this result may reflect that *Crocosphaera* take up fixed nitrogen during the day, but they fix nitrogen at night; leading to a certain balance between the two and the model reflects the average over the diel period. In addition, we note that typical values of nitrogen fixation and N content per cell from compiled data of *Trichodesmium*^48^ suggest that just fixing nitrogen at a typically observed rate can only support their growth of ~1 year^−1^ (see Supplementary Methods), significantly lower than the observed growth rate (Maximum growth rate of ~ 0.14 d^−1^ [ref.^48^]). This may indicate that other marine nitrogen fixers may actively use external fixed nitrogen and compete with non-nitrogen fixers.

In ecological models, this type of competition has not been considered, with the assumption that nitrogen fixers grow 100% diazotrophically anywhere in the modeled ocean^31,37,49^, likely based on forced diazotrophic growth in laboratory studies and low concentrations of fixed nitrogen^42,50–52^. However, our study shows that this may not always be true. Including the competitive aspect of nitrogen fixers may lead to a different model output; e.g. increased abundance of nitrogen fixing organisms and a decrease of non-nitrogen-fixing phytoplankton.

Where this type of competition happens and where not is still a question. This can be related to the concentration of fixed nitrogen and the time scale of its resource input. It can also be related to the resource ratio of fixed nitrogen to other nutrients (e.g. phosphorus and iron)^27,28^. To study that, chemostat cultures of *Crocosphaera* with various resource concentrations may be useful to further constrain the effects of N on nitrogen fixation. Additionally, CO_2_ might influence the growth^53^. Thus, to isolate the effect of nutrients on nitrogen fixation, CO_2_ concentration should be maintained stable or the concentration of CO_2_ must be measured frequently to clarify its daily fluctuation. Furthermore, growing *Crocosphaera* and non-nitrogen-fixing organisms e.g. *Synechococcus* spp. in chemostat culture and observing the flux of N under various resource nutrient concentrations can be useful. In this case, both the flux of N_2_ and dissolved N must be traced separately. Also, in the field, using labeled N (e.g. ^15^NH_4_^+^) and observing its fate would clarify the competition. To do that, a possible strategy might be isolating *Crocosphaera* with flow cytometry and measuring ^15^N with mass spectrometry or NanoSIMS.

### Dynamic and patchy ocean environment and meaning of storage and higher population

We point out that the ocean is highly patchy with numerous occasional upwelling regions^54,55^ and nutrient-depleted zones^56^, making the distribution of nutrients and plankton vary significantly throughout the ocean. Such patchiness may lead to chaotic distribution of nitrogen fixers and variable rates of nitrogen fixation. Within a smaller scale than generally modeled (e.g. 1° × 1°), there is a spectrum of nitrogen resources; in one place, there is active influx of fixed nitrogen while in other places there are zero or negative fluxes. In reality, such variable nitrogen fluxes may make it complex to determine whether *Crocosphaera* compete or help other organisms, which might be one cause of elusive nitrogen fixation rates^57^. Increasing the resolution of modeled grids to resolve eddies^58^ might be useful. Also, simply allowing fixed nitrogen uptake for modeled nitrogen fixers may cause some changes. Once these are combined, the ecological model may reproduce such observed patchiness in nitrogen fixation.

Given such a dynamic environment, our predicted increased cell densities and storage may be an advantage in sustaining species. When the environment shifts from P limitation to N limitation, with N storage, *Crocosphaera* may continue growing at a high rate until the storage is depleted. Higher population/larger niche may lead to a higher chance of survival as a group upon facing different zooplankton. Also, molecular studies suggest P scavenging by *Crocosphaera*^59,60^. Given these implications, it is surprising that *Crocosphaera* does not seem to accumulate excess phosphorus, the behavior often seen in other phytoplankton^61–64^. A recent modeling study showed that nitrogen fixers are limited by P in the Atlantic Ocean^65^. However, our model is calibrated to a strain from the Pacific Ocean (*Crocosphaera watsonii* PS0609A)^14^ where the nutrient is considered replete^65^. Lack of P storage might be a result of continued phosphorus repletion; a strain from the Atlantic Ocean might have P storing capacity. Alternatively, since the cellular space is limited, *Crocosphaera* might have chosen to use space for other purposes that are more important for their survival, such as photosystems, nitrogenase, or other nutrient storage.

## Conclusions

Based on a chemostat culture experiment, we have developed a model of *Crocosphaera* that combines uptake of available fixed nitrogen and nitrogen fixation. We have tested hypothetical conditions where the rate of nitrogen fixation is increased or is zero. The model indicates that increasing nitrogen fixation increases N storage, or their population, depending on nutrient limitation. We then simulate a simple ecological situation where *Crocosphaera* and non-nitrogen-fixing phytoplankton co-exist. The model suggests that *Crocosphaera* compete for N sources with non-nitrogen-fixing phytoplankton; increasing rate of nitrogen fixation can decrease the population of phytoplankton within a small timescale and distance. Our model results can be further tested by extensive laboratory measurements as well as field observations. Given the effect on N fluxes and phytoplankton population dynamics, reflecting the competitive aspects of *Crocosphaera* may be essential in predicting their roles, and those of other nitrogen fixers, in the changing environment.

## Methods

### Steady state model

In this section, we describe the model with equations, describing fundamental equations used in the steady state model and dynamic model (Additional details are in Supplementary Methods). Nomenclature with units, values used for adjustable and fixed parameters, initial values in the dynamic model, and used data from the chemostat experiment^14^ are provided in Tables S1–S7. The model includes three N molecules (*N*_*Growth*_, *N*_*Other*_, *N*_*Store*_) and two P molecules (*P*_*RNA*_, *P*_*Other*_). *N*_*Growth*_ includes growth related molecules rich in N, such as nitrogenase and proteins for photosynthesis and biosynthesis. Also, *P*_*RNA*_ (P contained in RNA) positively influences biomass production as well, since a large part of RNA is involved in protein synthesis^66^. In addition, we included N storage (*N*_*Store*_) for the cell to accumulate excess N. Other molecules in N and P are included in *N*_*Other*_ and *P*_*Other*_ respectively, representing basic need of N and P for maintaining cell viability^23,67^.

The model is manually parameterized to reproduce the chemostat culture conditions under which known concentrations of ammonium and phosphate were continuously added, when *Crocosphaera watsonii* PS0609A was grown^14^. The model is based on the minimum set of parameters, each of which has exclusive influence on model results, allowing us to narrow down the parameter values (Fig. S3; shown with a sensitivity study^68^). Chemostat cultures allow more realistic growth conditions, as nutrients can be gradually added at a steady rate, rather than in batch culture, where nutrient concentrations start high and are incrementally depleted during growth. Also, since the cellular growth rate can be controlled by adjusting the dilution rate, it allows us to model growth-rate dependency of cellular parameters. The study also offered a wide range of parameters (various elemental compositions and nitrogen fixation) for various specific growth rates and reported active nitrogen fixation despite the continuous addition of ammonium.

Once we parameterized the model, we manually changed the rate of nitrogen fixation to evaluate its effect on their biomass concentrations. In the environment, the interplay between competing strategies is likely influenced by competition with other microorganisms. These inter-organism interactions are challenging to reproduce under laboratory conditions, but undoubtedly influence growth rates due to competition for common resources.

The model resolves C, N and P fluxes and consists of coarse-grained macromolecules in N and P (Fig. 1). To simulate a chemostat culture where *Crocosphaera* grows under continuous addition of ammonium and phosphorus, we use fundamental balances of cellular quotas, cell densities, and dissolved nutrients. We recognize a distinct diurnal cycle of *Crocosphaera*^4–6^. However, to consider a steady state and to keep the model simple, we focus on the daily average of metabolisms and cellular quotas. Additionally, this decreases the number of free parameters.

1 $$\frac{1}{{C}_{Bio}}\frac{{dC}_{Bio}}{dt}={F}_{Photo}-\mu -{F}_{EPS}-{F}_{Res}$$

2 $$\frac{{dQ}_{N}}{dt}={V}_{N}+{F}_{Fix}^{N}-{\mu Q}_{N}-{F}_{DON}$$

3 $$\frac{{dQ}_{P}}{dt}={V}_{P}-{\mu Q}_{P}$$

4 $$\frac{dX}{dt}=\mu X-DX$$

5 $$\frac{d[EPS]}{dt}={F}_{EPS}X-D[EPS]$$

6 $$\frac{d[N]}{dt}=D({[N]}_{in}-[N])-{XV}_{N}$$

7 $$\frac{d[DON]}{dt}={F}_{DON}X-D[DON]$$

8 $$\frac{d[P]}{dt}=D({[P]}_{in}-[P])-{XV}_{P}$$

[Equation 1]~[Eq. 3] represent balances of cellular biomass C, *C*_*Bio*_, and cellular quotas of N and P quotas, *Q*_*N*_ and *Q*_*P*_, respectively. *C*_*Bio*_ is a balance of photosynthesis *F*_*Photo*_, growth *µ*, EPS (extracellular polymeric substances) excretion *F*_*Res*_ and respiratory loss *F*_*EPS*_ [Eq. 1]. Here, cellular excretion was not considered since it was not able to be constrained by the data. *Q*_*N*_ is balanced by nitrogen uptake *V*_*N*_, nitrogen fixation ${F}_{Fix}^{N}$, growth, and nitrogen excretion ${F}_{DON}$ [Eq. 2]. *Q*_*P*_ is simply a balance of uptake *V*_*P*_ and growth. [Eq. 4] shows that if cell density *X* is balanced by growth and dilution *D*. [Eq. 5]~[Eq. 8] are the balances of EPS and dissolved nutrients. EPS and DON ([*EPS*] and [*DON*] represents their concentrations respectively) are the balances of excretion (*F*_*EPS*_ and *F*_*DON*_ respectively)and dilution ([Eq. 5] and [Eq. 7]). It is possible that the uptake of DON may occur and we define ${F}_{DON}$ as net excretion that represents a balance of uptake and excretion of DON. Ammonium and phosphate are balances of nutrient flow and uptake ([Eq. 6] and [Eq. 8]), where [*j*] and [*j*]_in_ represent the concentration of *j* (here *N* (ammonium) or *P* (phosphate)) in the chemostat culture and incoming medium. We solve these equations assuming a steady state (d/dt = 0). A detailed solution of the model is in the Supplementary Material.

*C*_*Bio*_ does not appear in the steady state solution, since under the steady state, *dC*_*Bio*_/*dt* = 0. We use fluxes and quotas normalized by *C*_*Bio*_ to avoid repeated appearance of *C*_*Bio*_ following previous studies^23,24,67,69,70^. Total *Q*_*N*_ consists of growth related proteins *N*_*Growth*_, constant components, *N*_*other*_, which includes N for maintaining cells to be viable^71^, and N storage *N*_*store*_:

9 $${Q}_{N}={N}_{Growth}+{N}_{Other}+{N}_{Store}$$

*N*_*Growth*_ includes proteins for nitrogen fixation (nitrogenase), photosynthesis (photosystems), andother biosynthetic processes such as the synthesis of proteins, nucleic acids, carbohydrates and lipids. These proteins are shown to be significant in magnitude in proteomic studies^5,72,73^. We did not explicitly represent the molecular allocation to nutrient acquisition since it has been shown that the protein allocation to membrane transport is relatively small (less than 10%)^74^ and it has been predicted to be even smaller in a molecular allocation model^75^. However, we note that other models show the potential significance of molecules for nutrient-acquisition^70,76,77^ and thus more molecular and proteomic evidence would be needed. It has been known that cellular N contains a growth-rate-dependent part and a constant (maintenance/essential) part^23,78–81^, and the model reflects these; *N*_*Growth*_ for the former and *N*_*Other*_ for the latter. Also, storage molecules are recognized in various phytoplankton including *Crocosphaera*^6,82–85^, and we include this concept in the model as well. In the model, *N*_*Growth*_ linearly influences the rate of growth, photosynthesis and nitrogen fixation (see Supplementary Methods). *Q*_*P*_ consists of P in RNA *P*_*RNA*_ and P in other relatively constant molecules *P*_*Other*_, including phospholipids in cellular membranes.

10 $${Q}_{P}={P}_{RNA}+{P}_{Other}$$

Here *P*_*Other*_ includes a relatively constant P pool such as DNA and P in lipid membranes. RNA is observed to have a strong growth-rate dependency^63,66,86^, and to reflect that, we separate it from other P pools. Since a large part of RNA is involved in protein synthesis, in the model, it positively influences the rate of protein synthesis (see Supplementary Methods).

### Dynamic model

To test the effect of nitrogen fixation on biomass concentration in a more realistic environment, we simulated an ecological situation where *Crocosphaera* exists with other non-nitrogen-fixing phytoplankton. The model consists of fundamental balances of phytoplankton densities *X*_*i*_, zooplankton densities *X*_*zoo*_, cellular N and P quota of phytoplankton ${Q}_{N}^{i}$ and ${Q}_{P}^{i}$ respectively, and the concentration of inorganic nutrients in the culture [*j*]:

11 $$\frac{d{X}_{i}}{dt}={X}_{i}{\mu }_{i}-{X}_{Zoo}{G}_{i}$$

12 $$\frac{d{X}_{Zoo}}{dt}={X}_{Zoo}({G}_{Cro}+{G}_{Phy})-{m}_{2}{X}_{Zoo}^{2}$$

13 $$\frac{d{Q}_{N}^{i}}{dt}={V}_{N}^{i}-{\mu }_{i}{Q}_{N}^{i}+{F}_{Fix}^{N}$$

14 $$\frac{d{Q}_{P}^{i}}{dt}={V}_{P}^{i}-{\mu }_{i}{Q}_{P}^{i}$$

15 $$\frac{d[j]}{dt}={V}_{j}^{Cro}{X}_{Cro}+{V}_{j}^{Phy}{X}_{Phy}+{S}_{j}$$

where *i* is phytoplankton type (either *Crocosphaera* (*Cro*) or non-nitrogen-fixing phytoplankton (*Phy*)) and *j* is inorganic nutrient (either *N*, ammonium or *P*, phosphate), *G*_*i*_ represents grazing of phytoplankton *i* and *V* and *S* represents uptake and source terms. The term, *m*_2_ is a square mortality rate of zooplankton as used in a recent marine ecological model^37^. The equations are solved using the finite-difference method. *µ*_*i*_ is solved based on pseudo-steady state assumption where cellular components represent the steady state solution. ${F}_{Fix}^{N}$ is zero for non-nitrogen-fixing phytoplankton. *G*_*i*_ and ${V}_{j}^{i}$ are based on KTW (kill-the-winner) theory^87^ and Monod kinetics^88^, respectively (details for *µ*_*i*_, *G*_*i*_ and ${V}_{j}^{i}$ are in Supplementary Methods). The KTW method considered commonly observed active prey-switching behavior of zooplankton^89–91^, which are known to stabilize ecosystems^92,93^. Monod kinetics is a widely used equation for nutrient uptake, which well represents the general saturating relationship between nutrient uptake and concentration^94–96^.

## Supplementary information

Supplementary Information

## Data Availability

The models used in this study are freely available at https://github.com/ag105020/Croco2 (DOI: 10.5281/zenodo.2636804).

## References

[1] Zehr JP (2001). Unicellular cyanobacteria fix N_2_ in the subtropical North Pacific Ocean. Nature.

[2] Montoya JP (2004). High rates of N_2_ fixation by unicellular diazotrophs in the oligotrophic Pacific Ocean. Nature.

[3] Zehr JP (2011). Nitrogen fixation by marine cyanobacteria. Trends Microbiol..

[4] Mohr W, Intermaggio MP, LaRoche J (2010). Diel rhythm of nitrogen and carbon metabolism in the unicellular, diazotrophic cyanobacterium *Crocosphaera watsonii* WH8501. Environ. Microbiol..

[5] Saito MA (2011). Iron conservation by reduction of metalloenzyme inventories in the marine diazotroph *Crocosphaera watsonii*. Proc. Natl. Acad. Sci. USA.

[6] Dron A (2012). Light:dark (12:12 h) quantification of carbohydrate fluxes in *Crocosphaera watsonii*. Aquat. Microb. Ecol..

[7] Jacq, V., Ridame, C., L’Helguen, S., Kaczmar, F. & Saliot, A. Response of the unicellular diazotrophic cyanobacterium *Crocosphaera watsonii* to iron limitation. *PLoS ONE* **9**, e86749 (2014). 10.1371/journal.pone.0086749 PMC3897776 24466221

[8] Masuda T (2018). Diel regulation of photosynthetic activity in the oceanic unicellular diazotrophic cyanobacterium *Crocosphaera watsonii* WH8501. Environ. Microbiol..

[9] Gruber N, Galloway JN (2008). An Earth-system perspective of the global nitrogen cycle. Nature.

[10] Weber T, Deutsch C (2014). Local versus basin-scale limitation of marine nitrogen fixation. Proc. Natl. Acad. Sci. USA.

[11] Dekaezemacker J, Bonnet S (2011). Sensitivity of N_2_ fixation to combined nitrogen forms (NO_3_^−^ and NH_4_^+^) in two strains of the marine diazotroph *Crocosphaera watsonii* (Cyanobacteria). Mar. Ecol. Prog. Ser..

[12] Knapp AN, Dekaezemacker J, Bonnet S, Sohm JA, Capone DG (2012). Sensitivity of Trichodesmium erythraeum and Crocosphaera watsonii abundance and N_2_ fixation rates to varying NO_3_^−^ and PO_4_^3−^ concentrations in batch cultures. Aquat. Microb. Ecol..

[13] Knapp, A. N. The sensitivity of marine N_2_ fixation to dissolved inorganic nitrogen. *Front. Microbiol*. **3**, 374 (2012). 10.3389/fmicb.2012.00374 PMC3476826 23091472

[14] Masuda T, Furuya K, Kodama T, Takeda S, Harrison PJ (2013). Ammonium uptake and dinitrogen fixation by the unicellular nanocyanobacterium *Crocosphaera watsonii* in nitrogen-limited continuous cultures. Limnol. Oceanogr..

[15] Capone, D. G. Benthic nitrogen fixation. In: *Nitrogen Cycling in Coastal Marine Environments*. 85–123 (1988).

[16] Dixon R, Kahn D (2004). Genetic regulation of biological nitrogen fixation. Nat. Rev. Microbiol..

[17] Falkowski. ‘Enzymology of Nitrogen Assimilation’ in Nitrogen in the Marine Environment, eds Carpenter, E. J. and Capone, D. G. Academic Press, New York, NY. 839–868 (1983).

[18] Inomura K, Bragg J, Follows MJ (2017). A quantitative analysis of the direct and indirect costs of nitrogen fixation: a model based on *Azotobacter vinelandii*. ISME J..

[19] Inomura K, Bragg J, Riemann L, Follows MJ (2018). A quantitative model of nitrogen fixation in the presence of ammonium. PLOS ONE.

[20] Bühler T (1987). Control of dinitrogen fixation in ammonium-assimilating cultures of *Azotobacter vinelandii*. Arch. Microbiol..

[21] Schuster, S. & Fell, D. Modeling and simulating metabolic networks. In: Lengauer, T. (Ed.), Bioinformatics: From Genomes to Therapies. Wiley-VCH: Weinheim **2**, 755–805 (2007).

[22] Orth JD, Thiele I, Palsson BØ (2010). What is flux balance analysis?. Nat. Biotechnol..

[23] Geider RJ, Macintyre HL, Kana TM (1998). A dynamic regulatory model of phytoplanktonic acclimation to light, nutrients, and temperature. Limnol. Oceanogr..

[24] Pahlow M, Dietze H, Oschlies A (2013). Optimality-based model of phytoplankton growth and diazotrophy. Mar. Ecol. Prog. Ser..

[25] Nicholson DP, Stanley RHR, Doney SC (2018). A phytoplankton model for the allocation of gross photosynthetic energy including the trade-offs of diazotroophy. J. Geophys. Res. Biogeosciences.

[26] Tilman D (1977). Resource competition between plankton algae: An experimental and theoretical approach. Ecology.

[27] Dutkiewicz S, Ward BA, Monteiro F, Follows MJ (2012). Interconnection of nitrogen fixers and iron in the Pacific Ocean: Theory and numerical simulations. Global Biogeochem. Cycles.

[28] Ward BA, Dutkiewicz S, Moore CM, Follows MJ (2013). Iron, phosphorus, and nitrogen supply ratios define the biogeography of nitrogen fixation. Limnol. Oceanogr..

[29] Dutkiewicz S, Ward BA, Scott JR, Follows MJ (2014). Understanding predicted shifts in diazotroph biogeography using resource competition theory. Biogeosciences Discuss..

[30] Yoshikawa C, Coles VJ, Hood RR, Capone DG, Yoshida N (2013). Modeling how surface nitrogen fi xation in fl uences subsurface nutrient patterns in the North Atlantic. J. Geophys. Res..

[31] Stukel MR, Coles VJ, Brooks MT, Hood RR (2014). Top-down, bottom-up and physical controls on diatom-diazotroph assemblage growth in the Amazon River plume. Biogeosciences.

[32] Follett, C. L., Dutkiewicz, S., Karl, D. M., Inomura, K. & Follows, M. J. Seasonal resource conditions favor a summertime increase in North Pacific diatom–diazotroph associations. *ISME J*. **12**, 1543–1557 (2018). 10.1038/s41396-017-0012-x PMC5955908 29449611

[33] Moore LR, Goericke R, Chisholm SW (1995). Comparative physiology of *Synechococcus* and *Prochlorococcus*: influence of light and temperature on growth, pigments, fluorescence and absorptive properties. Mar. Ecol. Prog. Ser..

[34] Liu H, Landry MR, Vaulot D, Campbell L (1999). Prochlorococcus growth rates in the central equatorial Pacific: An application of the fmax approach. J. Geophys. Res..

[35] Fu FX, Warner ME, Zhang Y, Feng Y, Hutchins DA (2007). Effects of increased temperature and CO_2_ on photosynthesis, growth, and elemental ratios in marine *Synechococcus* and *Prochlorococcus* (Cyanobacteria). J. Phycol..

[36] Monteiro FM, Follows MJ, Dutkiewicz S (2010). Distribution of diverse nitrogen fixers in the global ocean. Global Biogeochem. Cycles.

[37] Dutkiewicz S (2015). Capturing optically important constituents and properties in a marine biogeochemical and ecosystem model. Biogeosciences.

[38] Pomeroy LP, Williams PL, Azam WF, Hobbie JE (2007). The microbial loop. Oceanography.

[39] Capone DG, Zehr JP, Paerl HW, Bergman B, Carpenter EJ (1997). *Trichodesmium*, a globally significant marine cyanobacterium. Science.

[40] Rodriguez, I. B. & Ho, T.-Y. Diel nitrogen fixation pattern of *Trichodesmium*: the interactive control of light and Ni. *Sci. Rep*. **4**, 4445 (2014). 10.1038/srep04445 PMC3963029 24658259

[41] Karl D (1997). The role of nitrogen fixation in biogeochemical cycling in the subtropical North Pacific Ocean. Nature.

[42] Hutchins DA (2007). CO_2_ control of *Trichodesmium* N_2_ fixation, photosynthesis, growth rates, and elemental ratios: Implications for past, present, and future ocean biogeochemistry. Limnol. Oceanogr..

[43] Capone DG, Ferrier MD, Carpenter EJ (1994). Amino acid cycling in colonies of the planktonic marine cyanobacterium Trichodesmium thiebautii. Appl. Environ. Microbiol..

[44] Shiozaki T (2018). Linkage Between Dinitrogen Fixation and Primary Production in the Oligotrophic South Pacific Ocean. Global Biogeochem. Cycles.

[45] Duce RA (2008). Impacts of atmospheric anthropogenic nitrogen on the open ocean. Science.

[46] Kodama T, Furuya K, Hashihama F, Takeda S, Kanda J (2011). Occurrence of rain-origin nitrate patches at the nutrient-depleted surface in the East China Sea and the Philippine Sea during summer. J. Geophys. Res. Ocean..

[47] Calil PHR, Richards KJ (2010). Transient upwelling hot spots in the oligotrophic North Pacific. J. Geophys. Res. Ocean..

[48] LaRoche J, Breitbarth E (2005). Importance of the diazotrophs as a source of new nitrogen in the ocean. J. Sea Res..

[49] Moore JK, Doney SC, Lindsay K (2004). Upper ocean ecosystem dynamics and iron cycling in a global three-dimensional model. Global Biogeochem. Cycles.

[50] Villareal TA (1990). Laboratory culture and preliminary characterization of the nitrogen-fixing Rhizosolenia-Richelia symbiosis. Mar. Ecol..

[51] Julio-José C-O, Stal LJ (1991). Diazotrophic growth of the unicellular cyanobacterium Gloeothece sp. PCC 6909 in continuous culture. J. Gen. Microbiol..

[52] Dron A (2012). Light-dark (12:12) cycle of carbon and nitrogen metabolism in Crocosphaera watsonii WH8501: relation to the cell cycle. Environ. Microbiol..

[53] Fu F-X (2008). Interactions between changing pCO_2_, N_2_ fixation, and Fe limitation in the marine unicellular cyanobacterium *Crocosphaera*. Limnol. Oceanogr..

[54] Lennert-Cody CE, Franks PJS (1999). Plankton patchiness in high-frequency internal waves. Mar. Ecol. Prog. Ser..

[55] Martin, A. P., Richards, K. J., Bracco, A. & Provenzale, A. Patchy productivity in the open ocean. *Global Biogeochem. Cycles* **16**, 1025 (2002).

[56] Hashihama F (2009). Macro-scale exhaustion of surface phosphate by dinitrogen fixation in the western North Pacific. Geophys. Res. Lett..

[57] Gruber N (2016). Elusive marine nitrogen fixation. Proc. Natl. Acad. Sci. USA.

[58] Clayton S (2017). Biogeochemical versus ecological consequences of modeled ocean physics. Biogeosciences.

[59] Dyhrman ST, Haley ST (2006). Phosphorus scavenging in the unicellular marine diazotroph *Crocosphaera watsonii*. Appl. Environ. Microbiol..

[60] Pereira, N., Shilova, I. N. & Zehr, J. P. Use of the high affinity phosphate transporter gene, *pstS*, as an indicator for phosphorus stress in the marine diazotroph *Crocosphaera watsonii* (*Chroococcales*, *Cyanobacteria*). *J. Phycol*. **55**, 752–761 (2019). 10.1111/jpy.12863 30929262

[61] Elrifi IR, Turpin DH (1985). Steady-state luxury consumption and the concept of optimum nutrient ratios: A study with phosphate nitrate limited *Selenastrum minutum* (Chlorophyta). J. Phycol..

[62] Healey FP (1985). Interacting effects of light and nutrient limitation on the growth rate of *Synechococcus linearis* (Cyanophyceae). J. Phycol..

[63] Rhee G-Y (1973). A continuous culture study of phosphate uptake, growth rate and polyphosphate in Scenedesmus sp. J. Phycol..

[64] Lin S, Litaker RW, Sunda WG (2016). Phosphorus physiological ecology and molecular mechanisms in marine phytoplankton. J. Phycol..

[65] Wang, W.-L., Moore, J. K., Martiny, A. C. & Primeau, F. W. Convergent estimates of marine nitrogen fixation. *Nature* **566**, 205–211 (2019). 10.1038/s41586-019-0911-2 30760914

[66] Daines SJ, Clark JR, Lenton TM (2014). Multiple environmental controls on phytoplankton growth strategies determine adaptive responses of the N:P ratio. Ecol. Lett..

[67] Pahlow M, Oschlies A (2009). Chain model of phytoplankton P, N and light colimitation. Mar. Ecol. Prog. Ser..

[68] Taniguchi DAA, Franks PJS, Poulin FJ (2014). Planktonic biomass size spectra: An emergent property of size-dependent physiological rates, food web dynamics, and nutrient regimes. Mar. Ecol. Prog. Ser..

[69] Pahlow M (2005). Linking chlorophyll-nutrietn dynamics to the Redfield N:C ratio with a model of optimal phytoplankton growth. Mar. Ecol. Prog. Ser..

[70] Pahlow M, Oschlies A (2013). Optimal allocation backs droop’s cell-quota model. Mar. Ecol. Prog. Ser..

[71] Scott M, Klumpp S, Mateescu EM, Hwa T (2014). Emergence of robust growth laws from optimal regulation of ribosome synthesis. Mol. Syst. Biol..

[72] Jahn M (2018). Growth of Cyanobacteria Is Constrained by the Abundance of Light and Carbon Assimilation Proteins. Cell Rep..

[73] Zavřel, T. *et al*. Quantitative insights into the cyanobacterial cell economy. *Elife* **8** (2019). 10.7554/eLife.42508 PMC6391073 30714903

[74] Christie-oleza, J. A., Sousoni, D., Lloyd, M., Armengaud, J. & Scanlan, D. J. Nutrient recycling facilitates long-term stability of marine microbial phototroph-heterotroph interactions. *Nat. Microbiol*. **2** (2017). 10.1038/nmicrobiol.2017.100 PMC5495174 28650444

[75] Klausmeier CA, Litchman E, Daufresne T, Levin SA (2004). Optimal nitrogen-to-phosphorus stoichiometry of phytoplankton. Nature.

[76] Burnap, R. L. Systems and photosystems: cellular limits of autotrophic productivity in cyanobacteria. *Front. Bioeng. Biotechnol*. **3** (2015). 10.3389/fbioe.2015.00001 PMC4299538 25654078

[77] Smith SL (2016). Flexible phytoplankton functional type (FlexPFT) model: Size-scaling of traits and optimal growth. J. Plankton Res..

[78] Garcia, N. S., Bonachela, J. A. & Martiny, A. C. Growth-dependent cell size controls interactions between nutrient supply and cellular elemental stoichiometry of marine *Synechococcus*. *ISME J* **10**, 2715–2724 (2016). 10.1038/ismej.2016.50 PMC5113841 27058506

[79] Liu H (1999). Cell cycle and physiological characteristics *Synechococcus* (WH7803) in chemostat cultu. Mar. Ecol. Prog. Ser..

[80] Claquin P, Martin-Jézéquel V, Kromkamp JC, Veldhuis MJW, Kraay GW (2002). Uncoupling of silicon compared with carbon and nitrogen metabolisms and the role of the cell cycle in continuous cultures of *Thalassiosira pseudonana* (Bacillariophyceae) under light, nitrogen, and phosphorus control. J. Phycol..

[81] Caperon J, Meyer J (1972). Nitrogen-limited growth of marine phytoplankton-I. Changes in population characteristics with steady-state growth rate. Deep Sea Res..

[82] Sterner RW, Chrzanowski TH, Elser JJ, George NB (1995). Sources of nitrogen and phosphorus Sources of nitrogen the growth supporting of in an oligotrophic and phytoplankton Canadian shield lake. Limnol. Ocean..

[83] Sterner, R. W. & Elser, J. J. Ecological Stoichiometry: the Biology of Elements from Molecules to the Biosphere. Princeton University Press: Princeton, NJ. (2002).

[84] Hall SR, Smith VH, Lytle DA, Leibold MA (2005). Constraints on primary producer N: P stoichiometry along N: P supply ratio gradients. Ecology.

[85] Li H, Sherman DM, Bao S, Sherman LA (2001). Pattern of cyanophycin accumulation in nitrogen-fixing and non-nitrogen-fixing cyanobacteria. Arch. Microbiol..

[86] Bremer, H. & Dennis, P. Modulation of chemical composition and other parameters of the cell by growth rate. In: Neidhardt, F. (eds). Escherichia coli and Salmonella typhimurium. Am. Soc. Microbiol.: Washington, DC, 1996. 1553–1569 (1996).

[87] Vallina SM, Ward BA, Dutkiewicz S, Follows MJ (2014). Maximal ingestion with active prey-switching: a kill-the-winner functional response and its effect on global species richness and biogeography. Prog. Oceanogr..

[88] Monod J (1949). The growth of bacterial cultures. Ann. Rev. Mar. Sci..

[89] Murdoch WW (1969). Switching in general predators: experiments on predator specificity and stability of prey populations. Ecol. Monogr..

[90] Kiørboe T, Saiz E, Viitasalo M (1996). Prey switching behaviour in the planktonic copepod Acartia tonsa. Mar. Ecol. Prog. Ser..

[91] Kalinkat, G., Rall, B. C., Vucic-Pestic, O. & Brose, U. The allometry of prey preferences. *PLoS ONE* **6**, e25937 (2011). 10.1371/journal.pone.0025937 PMC3187823 21998724

[92] Murdoch WW, Avery S, Smyth MEB (1975). Switching in predatory fish. Ecology.

[93] Morozov AY (2010). Emergence of Holling type III zooplankton functional response: Bringing together field evidence and mathematical modelling. J. Theor. Biol..

[94] Healey FP (1980). Slope of the Monod equation as an indicator of advantage in nutrient competition. Microb. Ecol..

[95] Milligan AJ (2004). Dynamics of Silicon Metabolism and Silicon Isotopic Discrimination in a Marine Diatom as a Function of *p*CO_2_. Limnol. Oceanogr..

[96] Follows MJ, Dutkiewicz S (2011). Modeling diverse communities of marine microbes. Ann. Rev. Mar. Sci..

