# Supplementary material for: Active nitrogen fixation by Crocosphaera expands their niche despite the presence of ammonium – A case study
Source: Sci Rep. 2019 Oct 21;9:15064. doi: 10.1038/s41598-019-51378-4 (PMC6803696; doi:10.1038/s41598-019-51378-4)
Supplement: Supplementary file 1 — Supplementary Information [file 41598_2019_51378_MOESM1_ESM.pdf]

# Active nitrogen fixation by *Crocosphaera* expands their niche despite the presence of ammonium – A case study

Keisuke Inomura, Takako Masuda and Julia M. Gauglitz

**Supplementary Information:** Supplementary Methods, Tables S1~S7, Figures S1~S3

# Supplementary Methods

## Additional details for the steady state model

**Determining molecular allocations in N and P.** Here we focus on the steady state solutions for the allocation of intracellular molecules; thus, the time variation of the molecular allocation is not described in the final form of the equations. Nitrogen fixation is our primary interest. The rate of nitrogen fixation $F_{Fix}^{N}$ is simply assumed proportional to availability of growth related enzymes $N_{Growth}$ with a rate coefficient $A_{Fix}^{N}$:

$$F_{Fix}^{N} = A_{Fix}^{N} N_{Growth} \qquad \text{[Eq. S1]}$$

Here $N_{Growth}$ includes proteins for photosynthesis (photosystems), nitrogen fixation (nitrogenase), and other biosynthesis; thus, increasing $N_{Growth}$ increases nitrogen fixation. The rate of photosynthesis has been described based on a frequently used saturating function[1,2]:

$$F_{Photo} = F_{Photo}^{max}\left(1 - e^{-I/I_0}\right) \qquad \text{[Eq. S2]}$$

where $F_{Photo}^{max}$ is maximum photosynthesis rate, $I$ is light intensity and $I_0$ is the light absorption and processing coefficient. Similar to previous models of photosynthetic cells[2,3], we have attributed $F_{Photo}^{max}$ to the flexible quota of nitrogen; here $N_{Growth}$, which includes photosynthetic proteins:

$$F_{Photo}^{max} = A_{Photo} N_{Growth} \qquad \text{[Eq. S3]}$$

In the chemostat study[4], saturated light is used. Thus, we assume that $e^{-I/I_0}$ is small (~ 0) and eliminating $F_{Photo}^{max}$ with [Eq. S2] and [Eq. S3], we obtain the following expression:

$$F_{Photo} = A_{Photo} N_{Growth} \qquad \text{[Eq. S4]}$$

To determine $F_{Nfix}$, we therefore need to specify $N_{Growth}$, which is obtained from a balance of $C_{Bio}$ [Eq. 1]. Under the steady state, the carbon produced by photosynthesis is balanced by growth (biomass production) $\mu$, respiration $F_{Res}$ and EPS excretion. Since growth

(biosynthesis) and nitrogen fixation require energy, which we assume is supported by respiration, $F_{Res}$ is represented as follows:

$$F_{Res} = A_{Res}^{\mu}\mu + A_{Res}^{Nfix} F_{Fix}^{N} \quad \text{[Eq. S5]}$$

Also, we assumed that EPS excretion is proportional to the growth rate since the cell production and EPS excretion are roughly proportional[5]:

$$F_{EPS} = A_{EPS}\mu \quad \text{[Eq. S6]}$$

Substituting [Eq. S4][Eq. S5][Eq. S1][Eq. S6] (in this order) into [Eq. 1] under the steady state leads to a solution for $N_{Growth}$ proportional to $\mu$:

$$N_{Growth} = \mu\, A_{Growth}$$

where

$$A_{Growth} = \frac{1 + A_{Res}^{\mu} + A_{EPS}}{A_{Photo} + A_{Res}^{Nfix} A_{Fix}^{N}} \quad \text{[Eq. S7]}$$

This equation indicates that increasing growth rates requires proportionately higher amounts of growth-related enzymes to produce new cells faster. In this model, rather than prescribing values to each parameter that makes up $A_{Growth}$, we directly assign a value to $A_{Growth}$, reducing the number of free parameters. Since this particular study focuses on the rate of nitrogen fixation, we do not prescribe values for $A_{Res}^{Nfix}$, $A_{Res}^{\mu}$ and $A_{Photo}$, but the above equations can be used to compute the rate of photosynthesis and respiration given appropriate values for these parameters. We note that this equation is empirically supported; cellular protein linearly increases with growth rate[6–8]. Here we define $Q_N^0$ as the sum of obtained $N_{Growth}$ and constant $N_{Other}$ representing proteins essential for the growth rate $\mu$:

$$Q_N^0 = N_{Growth} + N_{Other} \quad \text{[Eq. S8]}$$

If we eliminate $N_{Growth}$ with [Eq. S7] and [Eq. S8], the relationship becomes similar to the previous model representing a chemostat culture[9]:

$$Q_N^0 = \mu\, A_{Growth} + N_{Other} \qquad \text{[Eq. S9]}$$

which has been well fitted with a high resolution chemostat data[10]. Since RNA is related to protein production, the cell needs a certain amount of RNA to satisfy the protein production at growth rate of $\mu$:

$$P_{RNA} = A_{RNA}^{P}\, Q_N^0\, \mu \qquad \text{[Eq. S10]}$$

This relationship is supported by linearly increasing RNA/Protein with $\mu$ in laboratory studies[6,11]. If we eliminate $Q_N^0$ with [Eq. S9] and [Eq. S10] and then eliminate $P_{RNA}$ with [Eq. 10], a similar relationship to the previous model[9,12] is obtained:

$$Q_P = A_{Growth} A_{RNA}^{P} \mu^2 + N_{Other} A_{RNA}^{P} \mu + P_{Other} \qquad \text{[Eq. S11]}$$

**Determining nutrient limitation and computation of cellular population.** In the chemostat culture, either N or P is limiting the growth and cellular population since excess Fe is added[4]. To determine which nutrient is limited, we consider a balance of dissolved molecules in the culture [Eq. 6]~[Eq.8] and compute potential population of cells as well as intracellular balance of N and P [Eq. 2] and [Eq. 3]. The key equation for computing a population is the balance of $NH_4^+$ in the culture where its uptake depends on the population [Eq. 6]. However, we first need to clarify the uptake rate of per cellular C, $V_N$ . Based on [Eq. 2], under the steady state, the input of N into the cell ($V_N$ and $F_{Fix}^{N}$) is balanced by the consumption of N for growth ($\mu Q_N$ ) and DON excretion ($F_{DON}$):

$$V_N = -F_{Fix}^{N} + \mu Q_N + F_{DON} \qquad \text{[Eq. S12]}$$

Here we note that $Q_N$ linearly increases with the growth rate $\mu$. Thus, $V_N$ is quadratically related to $\mu$. From [Eq. 7] under the steady state, we find a relation between the rate of net DON excretion $F_{DON}$ and DON concentration $[DON]$:

$$F_{DON} = \frac{D[DON]}{X} \quad \text{[Eq. S13]}$$

In the chemostat culture, $[DON]$ is stable and apparently independent from $\mu$ (ref.[4]), leading us to assume that $[DON]$ is constant[4]. Also, under the steady state, from [Eq. 4]:

$$\mu = D \quad \text{[Eq. S14]}$$

When N is limited, there is no N storage, thus:

$$Q_N = Q_N^0 \quad \text{[Eq. S15]}$$

Substituting [Eq. S12][Eq. S13][Eq. S1][Eq. S7][Eq. S14][Eq. S15] (in this order) into [Eq. 6] under the steady state leads to a solution for $X_N$ ($X$ under N limitation):

$$X_N = \frac{[N]_{in} - [DON]}{Q_N^0 - A_{Fix}^N A_{Growth}} \quad \text{[Eq. S16]}$$

here we have assumed that $NH_4^+$ in the culture is mostly consumed due to N limitation based on the data of chemostat culture[4], thus, $[N]_{in} \gg [N]$. Similarly, considering the steady state, from [Eq. 3], [Eq. 8] and [Eq. S14], we obtain $X_P$ ($X$ under P limitation):

$$X_P = \frac{[P]_{in}}{Q_P} \quad \text{[Eq. S17]}$$

where we have similarly assumed that $PO_4^{3-}$ in the culture is mostly consumed due to P limitation based on the laboratory data[4], thus, $[P]_{in} \gg [P]$. When $X_N < X_P$, the culture is N limited and otherwise, it is limited by P.

**Determining other parameters.** Based on the data from the chemostat study[4], when N is limited, there is excess $PO_4^{3-}$, which is computed by solving [Eq. 3], [Eq. 4] and [Eq. 8] under the steady state:

$$[P] = [P]_{in} - X_N Q_P \quad \text{[Eq. S18]}$$

When P is limited, on the other hand, the data show $[N]_{in} \gg [N]$ indicating that N is accumulated in the intracellular storage $N_{Store}$:

$$N_{Store} = Q_N - Q_N^0 \quad \text{[Eq. S19]}$$

where

$$Q_N = \frac{[N]_{in} - [DON]}{X_P} + A_{Fix}^N A_{Growth}^N \quad \text{[Eq. S20]}$$

obtained by substituting [Eq. S12][Eq. S13][Eq. S1][Eq. S7][Eq. S14] (in this order) into [Eq. 6] (a similar process to obtaining [Eq. S16] with $X_P$ instead of $X_N$ ). Finally, since [*N*] is small, the total amount of nitrogen in the culture $[N_{Tot}]$ is approximated as the sum of cellular N and DON:

$$[N_{Tot}] = XQ_N + [DON] \quad \text{[Eq. S21]}$$

**Additional details for the dynamic model**

We estimate growth rate based on each nutrient quota following steady state equations [Eq. 9][Eq. 10][Eq. S7] [Eq. S9][Eq. S10] assuming $N_{Store} = 0$:

$$\mu_N^i = \frac{Q_N^i - N_{Other}^i}{A_{Growth}^i} \quad \text{[Eq. S22]}$$

$$\mu_P^i = \frac{-A_{RNA}^i N_{Other}^i + \sqrt{\left(A_{RNA}^i N_{Other}^i\right)^2 - 4A_{RNA}^i A_{Growth}^i \left(P_{Other}^i - Q_P^i\right)}}{2A_{RNA}^i A_{Growth}^i} \quad \text{[Eq. S23]}$$

and take the lower value.

We applied the KTW method for grazing $G_i$ (ref.[13]):

$$G_i = G_{max}\left(\frac{X_i^2}{X_{Cro}^2 + X_{Phy}^2}\right)\left(\frac{\left(X_{Cro} + X_{Phy}\right)^2}{K_G^2 + \left(X_{Cro} + X_{Phy}\right)^2}\right) \quad \text{[Eq. S24]}$$

where $G_{max}$ is the maximum grazing rate and $K_G$ is grazing-half-saturation constant. This method considered commonly observed active prey-switching behavior of zooplankton[14–16], which is known to stabilize ecosystems[17,18]. We use simple and commonly used Monod type nutrient uptake[19]:

$$V_j^i = V_{max,j}^i \frac{[j]}{[j] + K_j^i} \qquad \text{[Eq. S25]}$$

where $V_{max,j}^i$ and $K_j^i$ maximum nutrient uptake rate and a half saturation constant of nutrient $j$ for phytoplankton $i$.

**Calculation of diazotrophic growth rate of *Trichodesmium***

Here we show a simple calculation of the diazotrophic growth rate of *Trichodesmium* based on the compiled data[20]. The rate of nitrogen fixation is 0.1-10 (mol N mol $Chl^{-1}$ $h^{-1}$), chlorophyll per cell is 1.18-1.73 fmol $Chl^{-1}$ $cell^{-1}$, and nitrogen per cell is 3.6-70.4 pmol N $cell^{-1}$. We use values about in the middle of these ranges; i.e. nitrogen fixation rate of 5 (mol N mol $Chl^{-1}$ $h^{-1}$), chlorophyll per cell of 1.5 (fmol Chl $cell^{-1}$), and nitrogen per cell of 40 (pmol N $cell^{-1}$). Multiplying the first two numbers gives nitrogen fixation rate per cell of 7.5 (fmol N $cell^{-1}$ $h^{-1}$). Nitrogen per cell of 40 (pmol N $cell^{-1}$) is 40,000 (fmol N $cell^{-1}$). Dividing this number by the rate of nitrogen fixation per cell gives 12,000 (h), which equals 1.37 (year). This indicates that providing the N requirement for doubling a cell only by nitrogen fixation takes over 1 year, according to the compiled data.

**Table S1.** Nomenclature of the used symbols in the steady state model.

| Symbol | Definition | Unit |
|---|---|---|
| **State variables** | | |
| $[N]$ | Ammonium concentration | nmol N L$^{-1}$ |
| $[N_{Tot}]$ | Total nitrogen concentration | nmol N L$^{-1}$ |
| $[P]$ | Phosphate concentration | nmol P L$^{-1}$ |
| $C_{Bio}$ | Cellular biomass carbon | nmol cell$^{-1}$ |
| $Q_N$ | Cellular nitrogen quota (normalized by $C_{Bio}$) | mol N mol C$^{-1}$ |
| $Q_N^0$ | Essential nitrogen quota (normalized by $Q_C$ ) | mol N mol C$^{-1}$ |
| $Q_P$ | Cellular phosphorus quota (normalized by $C_{Bio}$) | mol P mol C$^{-1}$ |
| $N_{Growth}$ | Cellular nitrogen for growth | mol N mol C$^{-1}$ |
| $N_{Store}$ | Nitrogen storage | mol N mol C$^{-1}$ |
| $P_{RNA}$ | Phosphorus in RNA | mol P mol C$^{-1}$ |
| $X$ | Cellular density (in carbon) | nmol C L$^{-1}$ |
| | | |
| **Process rates** | | |
| $D$ | Dilution rate | d$^{-1}$ |
| $\mu$ | Growth rate | d$^{-1}$ |
| $F_{Photo}$ | Photosynthesis rate | mol C mol C$^{-1}$d$^{-1}$ |
| $F_{Res}$ | Respiration rate | mol C mol C$^{-1}$ d$^{-1}$ |
| $F_{EPS}$ | EPS excretion rate | mol C mol C$^{-1}$ d$^{-1}$ |
| $F_{Fix}^N$ | Nitrogen fixation rate | mol N mol C$^{-1}$ d$^{-1}$ |
| $F_{DON}$ | DON excretion rate | mol N mol C$^{-1}$ d$^{-1}$ |
| $V_N$ | Nitrogen uptake rate | mol N mol C$^{-1}$ d$^{-1}$ |
| $V_P$ | Phosphorus uptake rate | mol P mol C$^{-1}$ d$^{-1}$ |
| | | |
| **Parameters** | | |
| $A_{Fix}^N$ | Nitrogen fixation rate per $N_{Growth}$ | (mol N mol C$^{-1}$ d$^{-1}$) (mol N mol C$^{-1}$)$^{-1}$ |
| $A_{Res}^{\mu}$ | Coefficient for growth related respiration | mol C mol C$^{-1}$ |
| $A_{Res}^{Nfix}$ | Coefficient for respiration for nitrogen fixation | mol N$^{-1}$ mol C |
| $A_{Growth}$ | $N_{Growth}$ per growth rate | mol N mol C$^{-1}$ d |
| $A_{RNA}^P$ | Coefficient for P in RNA | mol P mol N$^{-1}$ d |
| $A_{Photo}$ | Photosynthesis rate per $N_{Growth}$ | mol N$^{-1}$ mol C d$^{-1}$ |

| | | |
|---|---|---|
| $F_{Photo}^{max}$ | Maximum photosynthesis rate | mol C mol $C^{-1}d^{-1}$ |
| $I_0$ | Light absorption and processing coefficient | µmol $m^{-2}$ $s^{-1}$ |
| $N_{Other}$ | Basal cellular nitrogen | mol N mol $C^{-1}$ |
| $P_{Other}$ | Basal phosphorus | mol P mol $C^{-1}$ |
| **Constants** | | |
| $[N]_{in}$ | Ammonium concentration in the incoming medium | nmol N $L^{-1}$ |
| $[DON]$ | DON concentration | nmol N $L^{-1}$ |
| $[P]_{in}$ | Phosphate concentration in the incoming medium | nmol P $L^{-1}$ |
| $I$ | Light intensity | µmol $m^{-2}$ $s^{-1}$ |
| $A_{EPS}$ | EPS excretion rate per growth rate | mol C mol $C^{-1}$ |
| **Others** | | |
| $t$ | Time | d |

**Table S2.** Additional symbols for the simple ecosystem model.

| Symbol | Definition | Unit |
|---|---|---|
| **State variables** | | |
| $[j]$ | Concentration of nutrient *j* | nmol C $L^{-1}$ |
| $Q_N^i$ | Cellular nitrogen quota of phytoplankton *i* (normalized by $C_{Bio}$) | mol N mol $C^{-1}$ |
| $Q_P^i$ | Cellular phosphorus quota of phytoplankton *i* (normalized by $Q_C$ ) | mol P mol $C^{-1}$ |
| $X_i$ | Cellular density of phytoplankton *i* (in carbon) | nmol C $L^{-1}$ |
| $X_{Zoo}$ | Cellular density of zooplankton (in carbon) | nmol C $L^{-1}$ |
| **Process rates** | | |
| $\mu_i$ | Growth rate of phytoplankton *i* | $d^{-1}$ |
| $V_j^i$ | Uptake rate of nutrient *j* for phytoplankton *i* | mol mol $C^{-1}$ $d^{-1}$ |
| $G_i$ | Grazing rate of phytoplankton *i* | $d^{-1}$ |
| **Parameters** | | |
| $S_j$ | Resource of nutrient *j* | nmol $L^{-1}$ $d^{-1}$ |
| $V_{max,j}^i$ | Maximum uptake rate of nutrient *j* for phytoplankton *i* | mol mol $C^{-1}$ $d^{-1}$ |
| $K_j^i$ | Half saturation constant of nutrient *j* for phytoplankton *i* | nmol C $L^{-1}$ |
| $G_{max}$ | Maximum grazing rate | $d^{-1}$ |
| $K_G$ | Grazing half saturation | nmol C $L^{-1}$ |
| $m_2$ | Square mortality rate | $d^{-1}$ nmol $C^{-1}$ L |
| **Others** | | |
| $i$ | Phytoplankton *i* (*Crocosphaera* or non-nitrogen-fixing phytoplankton). | - |
| $j$ | Nutrient *j* (Ammonium or phosphate). | - |

**Table S3.** Values used for parameters in the steady state model.

| Symbol | Value | Unit |
|---|---|---|
| $A_{Fix}^{N}$ | $7.00\times10^{-2}$ | (mol N mol $C^{-1}$ $d^{-1}$)<br>(mol N mol $C^{-1}$)$^{-1}$ |
| $A_{Growth}$ | *$1.56\times10^{-0}$ | mol N mol $C^{-1}$ d |
| $A_{RNA}^{P}$ | *$2.00\times10^{-1}$ | mol P mol $N^{-1}$ d |
| $N_{Other}$ | $1.28\times10^{-2}$ | mol N mol $C^{-1}$ |
| $P_{Other}$ | $7.05\times10^{-2}$ | mol P mol $C^{-1}$ |

* Half of the value is used for non-nitrogen-fixing phytoplankton.

Note: Once we specify the value of $A_{Growth}$, specific values for $A_{Photo}$, $A_{Res}^{\mu}$ or $A_{Res}^{Nfix}$ are not required to obtain the output in this specific study.

**Table S4.** Values used for the additional parameters for the simple ecosystem model.

| Symbol | Value | Unit |
|---|---|---|
| $S_{NH4}$ | $1.28 \times 10^{-1}$ | nmol N $L^{-1}$ $d^{-1}$ |
| $S_{PO4}$ | $3.55 \times 10^{-2}$ | nmol P $L^{-1}$ $d^{-1}$ |
| $V_{max,N}^{Cro}$ | #$2.56 \times 10^{-1}$ | mol N mol $C^{-1}$ $d^{-1}$ |
| $V_{max,P}^{Cro}$ | #$7.69 \times 10^{-2}$ | mol P mol $C^{-1}$ $d^{-1}$ |
| $K_N^{Cro}$ | $2.00 \times 10^{1}$ | nmol N $L^{-1}$ |
| $K_P^{Cro}$ | $1.00 \times 10^{1}$ | nmol N $L^{-1}$ |
| $G_{max}$ | $1.00 \times 10^{-0}$ | $d^{-1}$ |
| $K_G^2$ | $5.00 \times 10^{-0}$ | nmol C $L^{-1}$ |
| $m_2$ | $1.00 \times 10^{-2}$ | $d^{-1}$ nmol $C^{-1}$ L |

# Double the value is used for non-nitrogen-fixing phytoplankton.

**Table S5.** Values used for constants.

| Symbol | Value | Unit |
|---|---|---|
| $[N]_{in}$ | $5.00\times10^{4}$ | nmol N $L^{-1}$ |
| $[DON]$ | $1.29\times10^{-4}$ | nmol N $L^{-1}$ |
| $[P]_{in}$ | $2.00\times10^{4}$ | nmol P $L^{-1}$ |
| $A_{EPS}$ | $2.82\times10^{-1}$ | mol C mol $C^{-1}$ |

The values for $[N]_{in}$ and $[P]_{in}$ represent the actual concentrations in chemostat experiment[4]. The value for [*DON*] is the average measured value in Masuda et al., (2013) [4]. $A_{EPS}$ is based on the laboratory study where ~22% of POC is EPS[5]; EPS production ($A_{EPS}\mu$) / Cell C production ($\mu$) = 22% / (100% – 22%) = 0.282.

**Table S6.** Initial values in the simple ecological model

| Symbol | Value | Unit |
|---|---|---|
| $[N]$ | $6.00\times10^{-0}$ | nmol N $L^{-1}$ |
| $[P]$ | $3.20\times10^{-0}$ | nmol P $L^{-1}$ |
| $Q_N^{Cro}$ | $7.80\times10^{-2}$ | mol N mol $C^{-1}$ |
| $Q_N^{Phy}$ | $7.80\times10^{-2}$ | mol N mol $C^{-1}$ |
| $Q_P^{Cro}$ | $4.68\times10^{-2}$ | mol P mol $C^{-1}$ |
| $Q_P^{Phy}$ | $4.68\times10^{-2}$ | mol P mol $C^{-1}$ |
| $X_{Cro}$ | $1.00\times10^{0}$ | nmol C $L^{-1}$ |
| $X_{Phy}$ | $1.00\times10^{0}$ | nmol C $L^{-1}$ |
| $X_{Zoo}$ | $1.00\times10^{0}$ | nmol C $L^{-1}$ |

**Table S7.** Used data from the chemostat culture experiment[4].

| $\mu$ | POC | POP | $NH_4^+$ | $PO_4^{3-}$ | DON | Total N |
|---|---|---|---|---|---|---|
| ($d^{-1}$) | (μmol $L^{-1}$) | (μmol $L^{-1}$) | (μmol $L^{-1}$) | (μmol $L^{-1}$) | (μmol $L^{-1}$) | (μmol $L^{-1}$) |
| 0.1 | 287 | 12.2 | 0.059 | 0.052 | 13.4 | 83.9 |
| 0.15 | 289 | 18.0 | 0.025 | 0.049 | 13.1 | 69.3 |
| 0.2 | 259 | 12.8 | 0.015 | 5.7 | 13.2 | 69.1 |
| 0.25 | 192 | 10.4 | 0.008 | 7.1 | 11.4 | 69.3 |
| 0.3 | 155 | 13.2 | 0.009 | 8.8 | 12.9 | 62.1 |
| 0.35 | 103 | 8.01 | N.D. | 11.4 | 13.3 | 59.4 |

N.D. stands for not detectable.

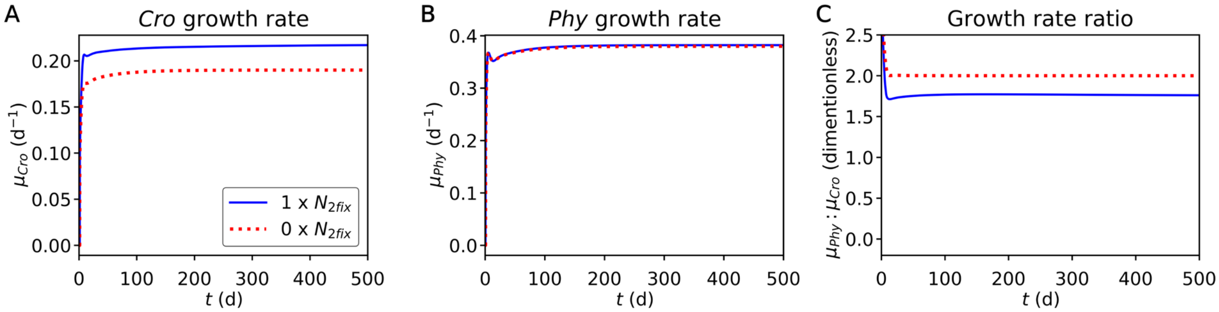


**Figure S1.** Growth rates and growth rate ratios from the simple ecosystem model. (A) Growth rates of *Crocosphaera*, $\mu_{Cro}$. (B) Growth rates of non-nitrogen-fixing phytoplankton, $\mu_{Phy}$. (C) The ratio of these two growth rates, $\mu_{Phy} : \mu_{Cro}$. Blue curves are the default run with nitrogen fixation. Red dotted curves are the run without nitrogen fixation.

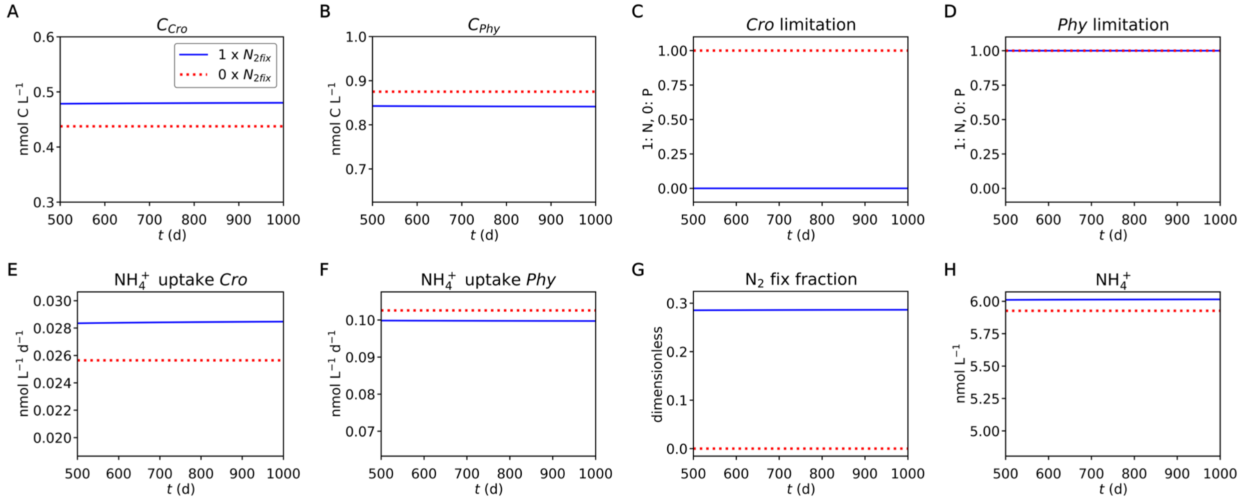


**Figure S2.** The results for the simple ecosystem model from day 500 to 1000. See Fig. 4, 5 for details. We have visually confirmed that the system has reached a steady state.

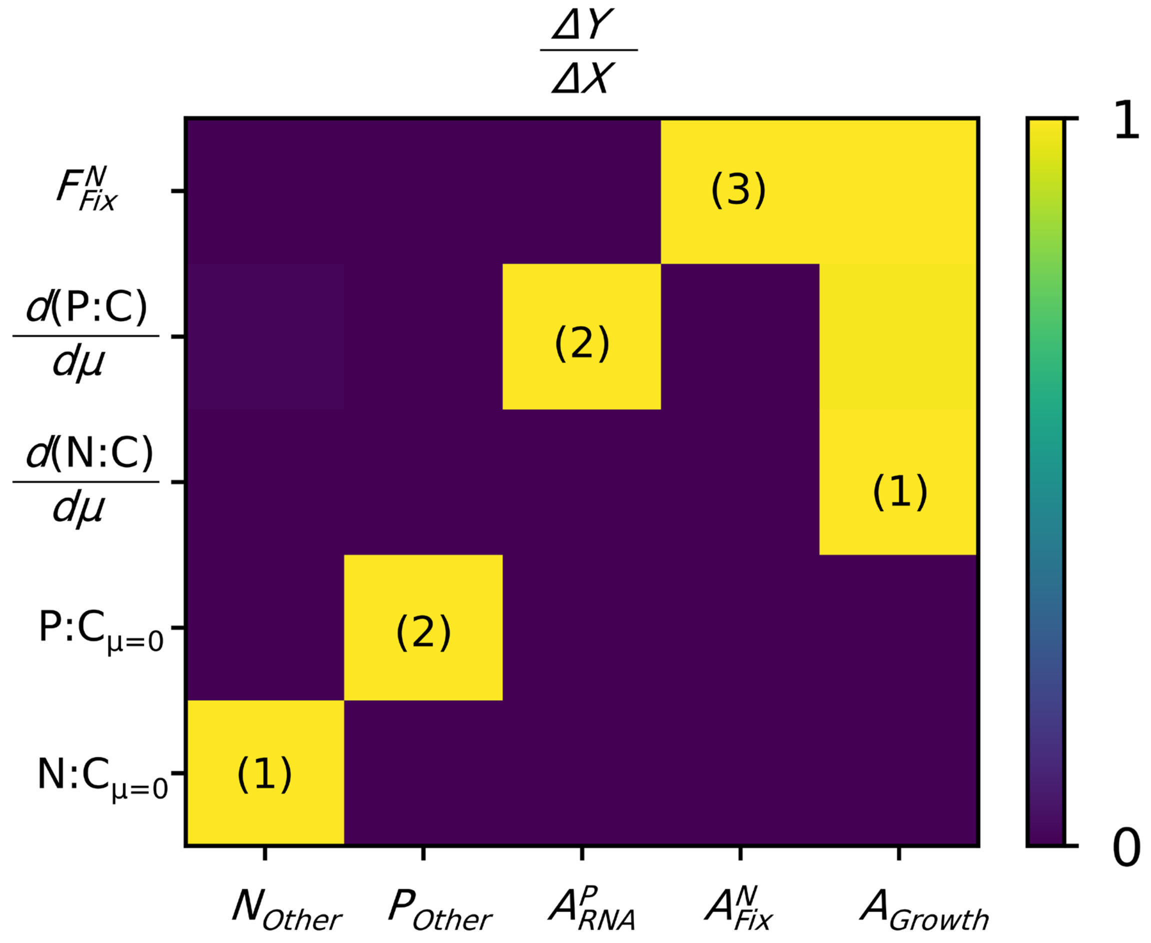


**Figure S3.** Sensitivity study of the steady state model. Color mesh indicates the change in the model output ($y$ axis) based on 1% change in each parameter ($x$ axis)[21]. The model outputs are at $\mu = 0.3$ ($d^{-1}$), thus N limited. In the $y$ axis, $\frac{d(\mathrm{N:C})}{d\mu}$ and $\frac{d(\mathrm{P:C})}{d\mu}$ are the slopes of N:C and P:C and $\mathrm{N:C}_{\mu=0}$ and $\mathrm{N:C}_{\mu=0}$ are the $y$-intercepts in N:C and P:C with relation to $\mu$. Here we consider N limitation; thus, $N_{Store} \sim 0$. Numbers in the mesh indicate the order of parameter determination explained as follows: (1) We first determine $N_{Other}$ and $A_{Growth}$ with N:C data. N:C in the data generally increase with $\mu$; thus $\mathrm{N:C} \sim \frac{d(\mathrm{N:C})}{d\mu}\mu + \mathrm{N:C}_{\mu=0}$. Since only $N_{Other}$ and $A_{Growth}$ have substantial influence on the slope and $y$-intercept respectively, these parameters are determined by fitting the model to N:C. (2) Next, we determine $P_{Other}$ and $A^{P}_{RNA}$ with P:C data. Similarly to N:C, $\mathrm{P:C} \sim \frac{d(\mathrm{P:C})}{d\mu}\mu + \mathrm{P:C}_{\mu=0}$. Here, only $P_{Other}$ influences the $y$-intercept but both $A_{Growth}$ and $A^{P}_{RNA}$ influence the slope. However, at this point, $A_{Growth}$ is already determined and only $A^{P}_{RNA}$ is left to fit the slope. (3) Finally, only $A^{N}_{Fix}$ is left to fit the rate of nitrogen fixation ($F^{N}_{Fix}$) and the value for $A^{N}_{Fix}$ is determined accordingly.

# References

1. Cullen, J. J. On models of growth and photosynthesis in phytoplankton. *Deep. Res.* **37**, 667–683 (1990).
2. Geider, R. J., Macintyre, H. L. & Kana, T. M. A dynamic regulatory model of phytoplanktonic acclimation to light, nutrients, and temperature. *Limnol. Oceanogr.* **43**, 679–694 (1998).
3. Pahlow, M. Linking chlorophyll-nutrietn dynamics to the Redfield N:C ratio with a model of optimal phytoplankton growth. *Mar. Ecol. Prog. Ser.* **287**, 33–43 (2005).
4. Masuda, T., Furuya, K., Kodama, T., Takeda, S. & Harrison, P. J. Ammonium uptake and dinitrogen fixation by the unicellular nanocyanobacterium *Crocosphaera watsonii* in nitrogen-limited continuous cultures. *Limnol. Oceanogr.* **58**, 2029–2036 (2013).
5. Sohm, J. A., Edwards, B. R., Wilson, B. G. & Webb, E. A. Constitutive extracellular polysaccharide (EPS) production by specific isolates of *Crocosphaera watsonii*. *Front. Microbiol.* **2**, 229 (2011).
6. Bremer, H. & Dennis, P. Modulation of chemical composition and other parameters of the cell by growth rate. In: Neidhardt F (eds). Escherichia coli and Salmonella typhimurium. Am. Soc. Microbiol.: Washington, DC, 1996. 1553–1569 (1996).
7. Rhee, G. Effects of N : P atomic ratios and nitrate limitation on algal growth, cell composition, and nitrate uptake. *Limnol. Oceanogr.* **23**, 10–25 (1978).
8. Felcmanová, K. *et al.* Carbon use efficiencies and allocation strategies in *Prochlorococcus marinus* strain PCC 9511 during nitrogen-limited growth. *Photosynth. Res.* **134**, 71–82 (2017).
9. Ågren, G. I. The C:N:P stoichiometry of autotrophs - Theory and observations. *Ecol. Lett.*

**7**, 185–191 (2004).

10. Elrifi, I. R. & Turpin, D. H. Steady-state luxury consumption and the concept of optimum nutrient ratios: A study with phosphate nitrate limited *Selenastrum minutum* (Chlorophyta). *J. Phycol.* **21**, 592–602 (1985).
11. Nicklisch, A. & Steinberg, C. E. W. RNA/protein and RNA/DNA ratios determined by flow cytometry and their relationship to growth limitation of selected planktonic algae in culture. *Eur. J. Phycol.* **44**, 297–308 (2009).
12. Scott, M., Mateescu, E. M., Zhang, Z. & Hwa, T. Interdependence of cell growth and gene expression: Originas and consequences. *Science* **330**, 1099–1103 (2010).
13. Vallina, S. M., Ward, B. A., Dutkiewicz, S. & Follows, M. J. Maximal ingestion with active prey-switching: A kill-the-winner functional response and its effect on global species richness and biogeography. *Prog. Oceanogr.* **120**, 93–109 (2014).
14. Murdoch, W. W. Switching in general predators: experiments on predator specificity and stability of prey populations. *Ecol. Monogr.* **39**, 335–354 (1969).
15. Kiørboe, T., Saiz, E. & Viitasalo, M. Prey switching behaviour in the planktonic copepod Acartia tonsa. *Mar. Ecol. Prog. Ser.* **143**, 65–75 (1996).
16. Kalinkat, G., Rall, B. C., Vucic-Pestic, O. & Brose, U. The allometry of prey preferences. *PLoS ONE* **6**, (2011).
17. Murdoch, W. W., Avery, S. & Smyth, M. E. B. Switching in predatory fish. *Ecology* **56**, 1094–1105 (1975).
18. Morozov, A. Y. Emergence of Holling type III zooplankton functional response: Bringing together field evidence and mathematical modelling. *J. Theor. Biol.* **265**, 45–54 (2010).
19. Monod, J. The growth of bacterial cultures. *Ann. Rev. Mar. Sci.* **3**, 371–394 (1949).

20. LaRoche, J. & Breitbarth, E. Importance of the diazotrophs as a source of new nitrogen in the ocean. *J. Sea Res.* **53**, 67–91 (2005).

21. Taniguchi, D. A. A., Franks, P. J. S. & Poulin, F. J. Planktonic biomass size spectra: an emergent property of size-dependent physiological rates, food web dynamics, and nutrient regimes. *Mar. Ecol. Prog. Ser.* **514**, 13–33 (2014).
